# Intestinal organoid co-culture protocol to study cell competition *in vitro*

**DOI:** 10.1016/j.xpro.2021.101050

**Published:** 2021-12-16

**Authors:** Sanne M. van Neerven, Rana Ramadan, Milou S. van Driel, David J. Huels, Louis Vermeulen

**Affiliations:** 1 Laboratory for Experimental Oncology and Radiobiology, Center for Experimental and Molecular Medicine, Cancer Center Amsterdam and Amsterdam Gastroenterology Endocrinology and Metabolism, Amsterdam University Medical Centers, Meibergdreef 9, 1105 Amsterdam, the Netherlands; 2 Oncode Institute, Meibergdreef 9, 1105 Amsterdam, the Netherlands

**Keywords:** Cell Biology, Cell culture, Flow Cytometry/Mass Cytometry, Cell-based Assays, Cancer, Microscopy, Organoids

## Abstract

Intestinal organoid cultures are a powerful tool to study epithelial cells *in vitro*, as they are able to proliferate and differentiate into all cell lineages observed *in vivo*. Co-culturing organoids with distinct genetic backgrounds provides an excellent approach to study contact dependent and independent interactions between healthy and mutant epithelial intestinal cells. Here, we provide 2D and 3D approaches to mouse organoid co-cultures using fluorescently labeled organoids and demonstrate the analysis of these co-cultures using flow cytometry and microscopy-based approaches.

For complete details on the use and execution of this profile, please refer to van Neerven et al., 2021.

## Highlights

• Protocol for 2D and 3D intestinal organoid co-culture approaches
• Generation and tracing of fluorescently labeled organoids over time
• Applying microscopy and flow cytometry techniques for co-culture analysis
• Study the interactions between genetically distinct cell populations

## Graphical abstract

**Figure:**
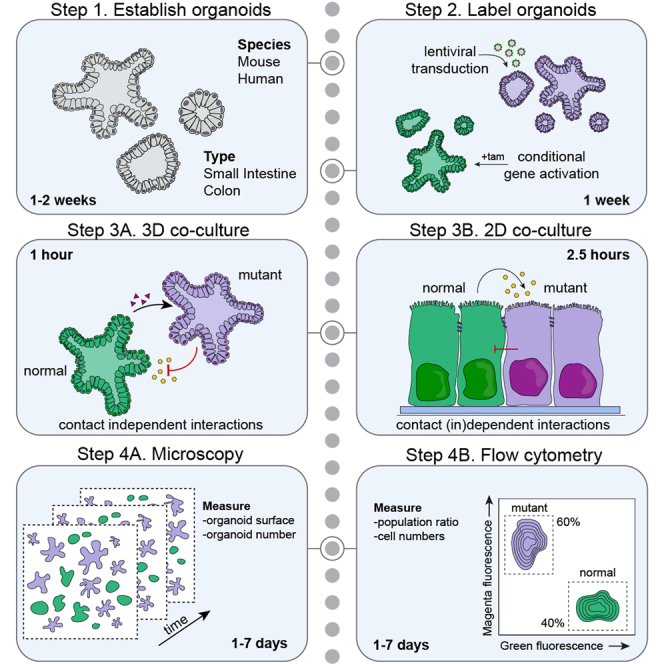

## Before you begin

**Figure 1:**
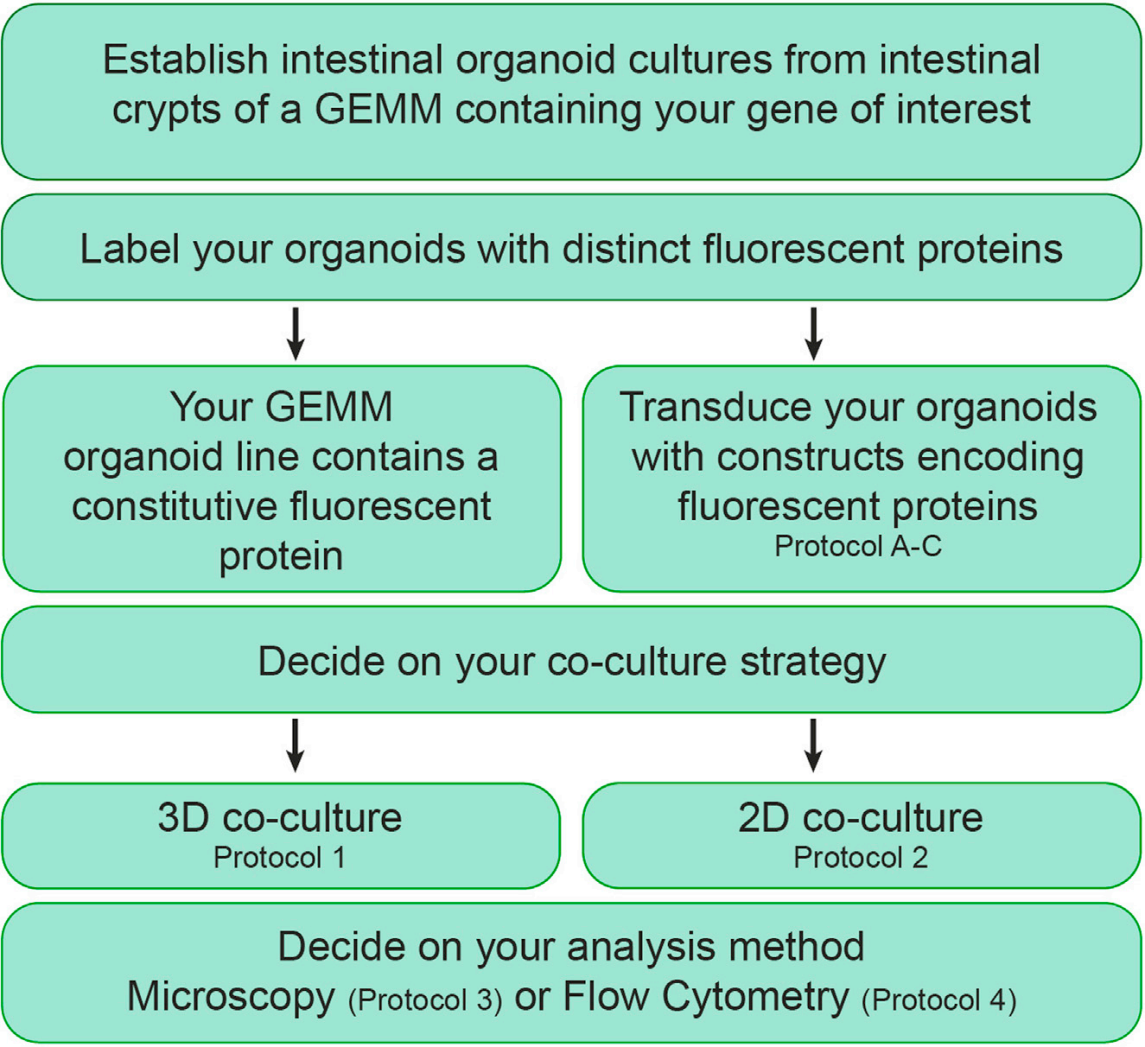
Workflow and options for co-cultures using intestinal organoids. GEMM; genetically engineered mouse model

This protocol describes the generation of fluorescently labeled intestinal organoid cultures for 2D and 3D co-culture approaches that can be analyzed using microscopy imaging and/or flow cytometry analysis. Using these techniques, a schematic workflow of this protocol is provided as Figure 1.

### Establish organoid cultures

This protocol describes co-culture techniques for genetically distinct intestinal organoids. To perform these experiments, it is essential to establish organoid cultures from crypt isolations of the human or murine intestine. Normal and mutant murine organoid cultures can be established by isolating crypts from wild type mice or genetically engineered mouse models (GEMM) that carry a (conditional) knockout for your gene of interest. Human organoid cultures are generated from surgically resected intestinal tissue or endoscopic biopsies. Detailed protocols on the isolation and generation of murine and human organoid cultures have been extensively described elsewhere (Sato et al., 2009, 2011). In addition, these protocols provide a detailed description of the culture medium conditions for murine and human organoids derived from both the small intestine and colon. In the protocol below, we demonstrate co-culture methods using mouse intestinal organoids derived from the proximal small intestine, but these protocols are also applicable for murine and human organoids derived from different intestinal regions.

**CRITICAL:** Before starting with your organoid co-cultures, validate the correct genotype of your organoids. In case of inducible gene activation/loss, make sure to check complete recombination of mutant alleles within your organoid population.

***Note:****In vitro* recombination of the mutant alleles of the gene of interest, rather than recombining *in vivo*, would be beneficial as the parental ‘unrecombined’ line can function as a control. This allows for an optimal study of competition between wild type and mutant organoid cultures.

***Alternatives:*** Mutations can be introduced *in vitro* using gene editing techniques such as CRISPR-Cas9. An example of a detailed protocol on CRISPR-Cas9 gene editing in organoids can be found elsewhere (Schwank and Clevers, 2016). When opting for CRISPR-Cas9 gene editing approaches one should take into account the extensive validation of the mutation which is often accompanied by the time-consuming generation of single-cell derived organoid clones to generate a homogeneously edited culture.

### Decide on fluorescent labeling technique

In order to trace organoids in co-culture, genetically distinct cultures can be visualized using fluorescent proteins. Fluorescently labeled organoid cultures can be generated using (1) cassettes encoding fluorescent proteins already present in your GEMM, or (2) transduction of lentiviral vectors encoding fluorescent proteins.

**CRITICAL:** In case your GEMM carries a fluorescent cassette, ensure the locus in which the cassette is introduced is constitutively expressed along all cell types, such as e.g., *Rosa26*. In addition, if the fluorescent cassette must be conditionally activated, please ensure full recombination to enable a 100% labeled population.

If your GEMM does not carry such a cassette, fluorescent constructs can be introduced in your organoid cultures by lentiviral transduction. The protocol to generate viral particles (A), transduce (B) and sort (C) your organoids is described below (Protocol A-C).

**CRITICAL:** When deciding on the fluorophores you want to use, consider the excitation/emission spectra of the fluorophores to avoid overlapping spectra. BD Biosciences provides a helpful online spectral viewer that can aid the choice of fluorophores (https://www.bdbiosciences.com/en-us/resources/bd-spectrum-viewer. In addition, ensure that your detection machines (Flow Cytometer/Microscope) have the appropriate lasers to detect the fluorophores. For the protocol described below, we make use of mVenus (excitation: 515, emission: 527) and mCherry (excitation: 587, emission: 610) fluorophores that generate clearly distinguishable, non-overlapping populations.

***Note:*** The use of nuclear localized fluorophores can help with a better visualization of distinct cells as most segmentation analysis tools function more appropriately on labeled nuclei compared to membrane stained cells.

**CRITICAL:** For all co-cultures, it is critical to compare the experimental co-culture (wild type/mutant) with a ‘control’ co-culture consisting of wild type organoids in 2 distinct fluorescent colors (wild type/wild type). Take this into account when labeling your organoids

### Decide on your co-culture strategy

The protocols below describe the use of both 2D and 3D co-culture approaches, and the choice for either one of these approaches is dependent on your research question. 3D organoid cultures are a valuable asset for research on the intestine in health and disease as they reflect the intestinal cell composition and homeostasis as observed *in vivo*. 3D co-cultures can be elegantly used to e.g., study influences of mutant cells on normal intestinal homeostasis (van Neerven et al., 2021). Limitations of 3D cultures are the inaccessibility of the lumen and the difficulty of imaging superimposed organoids. 2D organoid cultures help in overcoming these limitations and provide the opportunity to generate polarized monolayers with the luminal side exposed. As such, 2D layers provide an excellent method to study cell-cell contact dependent interactions. It is important to note that the distribution of cell types is different between cells grown on collagen and in Matrigel (Ramadan et al., 2021).

### Decide on your experimental readout

To study the competition between fluorescently labeled organoid populations both visual, microscopy-based and quantitative, flow cytometry-based approaches can be used. For 3D co-cultures, microscopy enables visual assessment regarding organoid size and shape. For both 2D and 3D cultures, microscopy generates two-dimensional images that can be used to measure the size of individual cells/organoids or can be used to determine the collective surface occupation of each labeled population within the culture. However, in case co-cultured organoids have distinct growth modes or cell shapes flow cytometry analysis can also be used to measure absolute cell numbers instead of cell surface. When analyzing your co-cultures at different timepoints, information regarding expansion rates can be acquired. In addition, both 2D and 3D organoid co-cultures can be expanded over multiple passages to assess differences in clonogenic capacity. Depending on your research question, either one of the two experimental readouts can be used. However, for a complete analysis of your co-culture, we advise to use both microscopy and flow cytometry-based techniques.

### Protocol A. Generation of lentiviral particles

**Timing: 5 days**

**CRITICAL:** All experiments involving lentiviral particles must be performed in a biosafety level 2 (BSL 2) lab. Please take care in following BSL 2 regulations on experimental handling and discarding of plastics, cells and media.

This protocol describes the generation of lentiviral particles using lipofectamine transfection and the 3^rd^ generation lentiviral packaging system. This packaging system enables efficient viral particle production from all 3^rd^ generation lentiviral plasmids. Here, we use plasmids encoding mVenus (LeGO-V2, Addgene #27340) and mCherry (LeGO-C2, Addgene #27339) fluorescent proteins.

**CRITICAL:** This protocol cannot be used for packaging of 2^nd^ generation plasmids.

***Alternatives:*** an alternative protocol for the generation of viral particles using Polyethylenimine (PEI) as transfection agent, followed by transduction of intestinal organoids is described elsewhere (De Van Lidth Jeude et al., 2015).

#### Day 1

**Timing: 30 min**

1. Pre-warm a bottle of DMEM supplemented with 10% FCS to 37°C.
2. Split a 70%–80% confluent 10 cm plate of HEK293T cells 1:4 into fresh 10 cm plates to ensure ∼70% confluency 24 h later. Use 1 plate per lentivirus.

#### Day 2

**Timing: 30 min**

3. Prepare the following transfection mixtures in 15 mL tubes. Per reaction:
  a. Mix 40 μL lipofectamine 2000 and 1.5 mL of OptiMEM. Incubate for 5 min at room temperature (20°C–22°C).
  b. Mix packaging plasmids and the vector of your choice in 1.5 mL OptiMEM according to the following Table 1:

**Table 1:** components of the packaging mixture

Plasmid	Amount
pMD2.G (envelope)	2.5 μg
pMDLg/pRRE (packaging)	3.5 μg
pRSV-Rev (packaging)	2.0 μg
Vector of choice	8.0 μg

***Note:*** The same plasmid mixture can be used to generate lentiviral particles to transduce human organoids.

4. Combine DNA and lipofectamine mixture (a total of 3 mL per reaction). Incubate for at least 20 min at room temperature.

***Note:*** The transfection mixture is stable for 6 h.

5. In the meantime, remove the DMEM (supplemented with 10% FCS) from 10 cm plates containing 70% confluent HEK293T cells.
6. Carefully wash the plate with PBS to remove any remaining serum.
7. Add 12 mL pre-warmed OptiMEM per 10 cm plate, place them back in the incubator until the transfection mixture is ready.
8. Add the 3 mL transfection mixture to the 10 cm plates (end volume of 15 mL) and incubate overnight.

#### Day 3

**Timing: 5 min**

9. Remove the OptiMEM and replace it with 8 mL pre-warmed DMEM;10%FCS.

#### Day 4

**Timing: 5 min**

10. Collect the medium after 24 h and temporarily store the medium containing viral particles at 4°C.
11. Add fresh 8 mL pre-warmed DMEM;10%FCS and place back in the incubator.

#### Day 5

**Timing: 1 h**

12. Collect the medium after 48 h and add to the tube containing the 24 h harvest.

***Note:*** Successful transfection can be validated by checking for expression of your fluorophore in the HEK293T cells under a fluorescent microscope.

13. In order to remove any dead cells from the harvest, spin the tube in a centrifuge for 4 min 210×*g*, continue with the supernatant.
14. Filter the supernatant through a 0.45 μm filter using a 50 mL syringe and transfer the supernatant to an Amicon 100 kDalton centrifugal filter.

***Note:*** The 100 kDalton Amicon centrifugal filters are contained within 50 mL tubes. You can load a maximum of 15 mL medium containing lentiviral particles on top of the filter. If more medium needs to be concentrated, carefully lift up the filter, discard the flow-through, place back the filter, load more medium on top of the filter and centrifuge again.

15. Concentrate the virus by centrifuging the Amicon filters for 30 min on 6750×*g* at 4°C.
16. Collect the concentrated virus in the top of the filter and transfer to a cryogenic vial.
17. Store the virus in the cryogenic vial or aliquot into smaller containers to prevent freeze/thaw cycles, store at −80°C.

***Note:*** Viral particles collected from a 10 cm plate in 16 mL (2 harvests of 8 mL) will be concentrated into approximately 200–300 μL.

**Pause Point:** Lentiviral particles can be stored at −80°C for at least a year, make sure to avoid freeze-thaw cycles. In our experience, lentiviral particles can even be used for up to two years, if storing conditions are maintained properly.

### Protocol B. Transduction of organoids

**Timing: 2 days**

**CRITICAL:** All experiments involving lentiviral particles must be performed in a biosafety level 2 (BSL 2) lab. Please take care in following BSL 2 regulations on experimental handling and discarding of plastics, cells and media.

**Figure 2:**
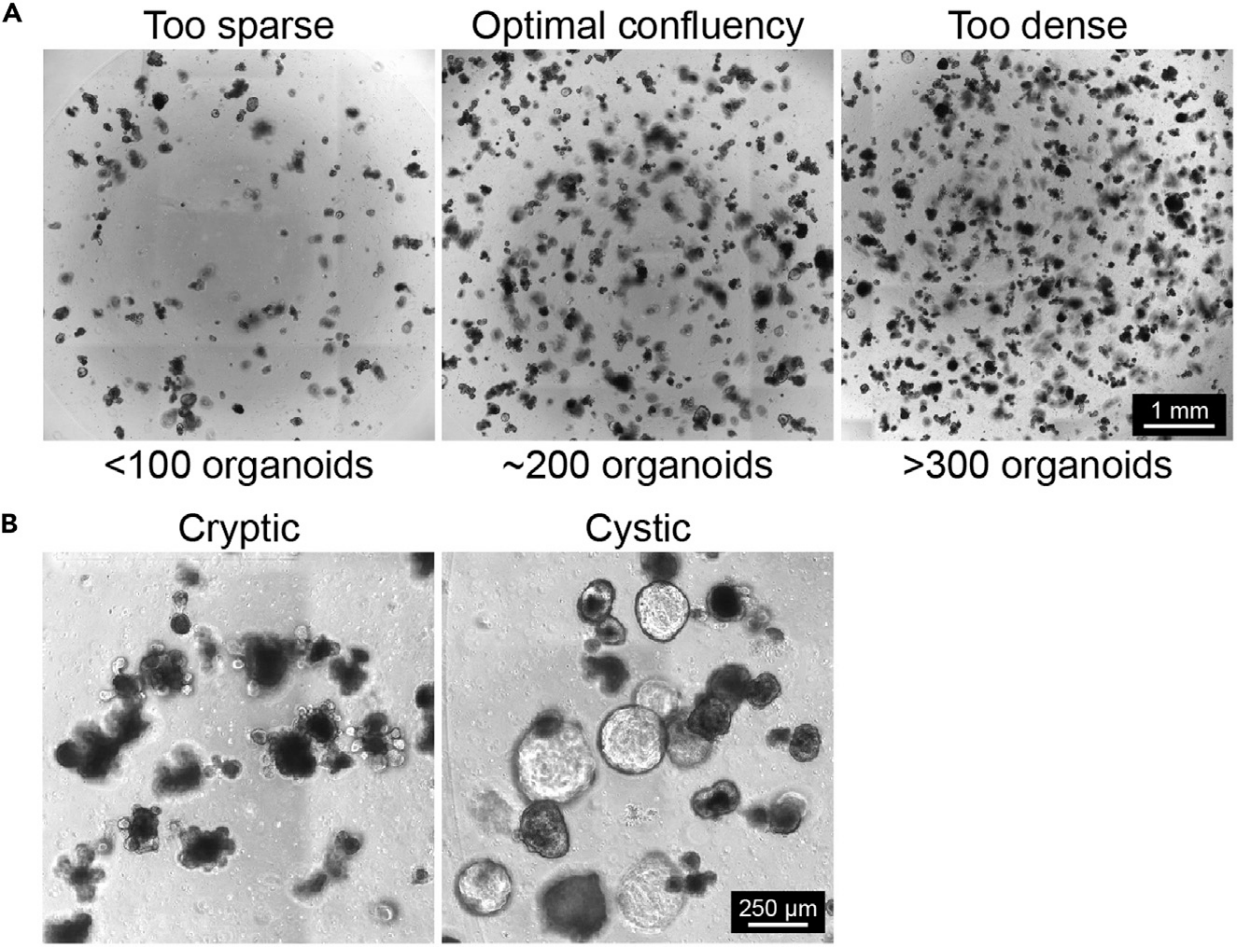
Optimal conditions for organoid transduction (A) visual illustration of organoids plated in different densities, the middle panel shows optimal confluency for transduction. (B) Three-day treatment with 10 μM CHIR-99021 changes organoid shape from cryptic to cystic.

***Note:*** Per transduction you need around 2 full wells of organoids growing in 50 μL Matrigel domes grown in 24-well plates. Figure 2A shows an example of a ‘full’ well of organoids, and wells that have been seeded too sparsely or too dense. At least 3 days before the transduction add 10 μM CHIR-99021 to an organoid medium containing mEGF, Noggin and R-spondin (ENR medium) to increase the stem cell-like state of your organoids. See Table 2 in “materials and equipment” for the correct preparation of the ENR medium. CHIR-99021 is an inhibitor of Glycogen Synthase Kinase 3β (GSK3β), an essential component of the construction complex responsible for the degradation of β-catenin. Inhibition of GSK3β results in nuclear translocation of β-catenin and transcription of Wnt pathway target genes (Naujok et al., 2014). Figure 2B gives an example of a well containing wild type organoids cultured with and without CHIR-99021, as a result of the CHIR, your organoids grow cystic. Please ensure you maintain at least 1 full well of untransduced organoids to serve as a negative control to set your FACS gating.

***Alternatives:*** Instead of CHIR-99021, the Wnt pathway can also be stimulated by supplementation of Wnt3a to the ENR medium, either by Wnt3a conditioned medium made by a producer line or by recombinant Wnt3a protein.

**Table 2:** Components of standard organoid medium

Component	Final concentration	Per bottle (500 mL)	Per 100 mL	Storage temperature
Advanced DMEM/F-12	n/a	450 mL	90 mL	4°C
N-2 Supplement (100×)	1×	5 mL	1 mL	−20°C
B-27 Supplement (50×)	1×	10 mL	2 mL	−20°C
GlutaMAX (100×)	1×	5 mL	1 mL	4°C
HEPES buffer (1 M)	10 mM	5 mL	1 mL	4°C
Antibiotic-Antimycotic (100×)	1×	5 mL	1 mL	−20°C
N-Acetyl-L-cysteine (1 M)	1 mM	1 mL	0.2 mL	−20°C

#### Day 1

**Timing: 1.5 h**

18. Check your organoids under the microscope to assess whether they are suited for transduction. Organoids should be cystic (Figure 2B).
19. Remove the ENR medium
20. Collect the Matrigel containing the organoids in 500 μL ice-cold Cell Recovery Solution (Use 2 full wells for every transduction with lentiviral particles) and transfer to a 15 mL tube.
21. Pipette the mixture vigorously up and down to generate small clumps of organoid cells

**CRITICAL:** A single cell suspension of organoid cells does not grow out as efficiently.

22. Incubate on ice for 30 min to dissolve the Matrigel.
23. Centrifuge for 4 min at 480×*g* to spin down the organoids. A clean, small pellet should be visible, remove the supernatant.

***Note:*** Sometimes a small layer of undissolved Matrigel can be visible directly above the cell pellet, if so, you can carefully remove this layer while keeping the cell pellet intact.

24. Mix transduction medium. Per transduction with lentiviral particles you need:
  a. 250 μL ENR medium
  b. 8 μg/mL Polybrene
  c. 10 μM CHIR-99021
  d. 10 μM Y-27632 dihydrochloride (ROCK inhibitor)
25. Add the transduction mixture to your organoids and pipette up and down before transferring it to a 48-well plate. Use 1 well per lentiviral transduction
26. Add 20 μL concentrated virus per 48-well with to be transduced organoids.

***Note:*** The amount of virus needed depends on the efficiency of lentiviral particle generation in Protocol A. Under standard conditions, in our hands 20 μL virus should be sufficient. Using this amount, we aim to acquire 10%–30% positive cells per transduction. If transduction is not efficient (less than 10% positive cells), please refer to the troubleshooting section for optimization.

27. Centrifuge the plate for 30 min at 1080×*g* at 32°C to increase the contact between the cells and the virus.
28. Store the plate in the incubator at 37°C containing 5% CO_2_ overnight (16–24 h), in the absence of Matrigel.
29. Pre-warm a 24-well plate in the incubator for the day after.

#### Day 2

**Timing: 30 min**

Before you start: Thaw an aliquot of Matrigel on ice 1 h prior to use.

30. Check your organoids under the microscope.

***Note:*** You will observe a mixture between attached and floating clumps of cells. All cells within the well should be collected.

31. Collect the transduction medium containing floating organoids in a 15 mL tube.
32. Wash the 48-well plate with ice-cold PBS to detach the attached organoids, transfer to the same 15 mL tube. Repeat this step three times.
33. Centrifuge for 4 min at 480×*g* to spin down the organoids, a small pellet should be visible.
34. Remove the supernatant and wash the organoids with 1 mL PBS.
35. Centrifuge for 4 min at 480×*g*, completely remove the supernatant.
36. Resuspend the pellet in 100 μL Matrigel and divide over 2 wells (50 μL each) in the pre-warmed 24 well plate.
37. Turn the plate upside down inside the incubator and incubate for 5–10 min to solidify the Matrigel.
38. Prepare ENR medium containing 10 μM CHIR-99021 and 10 μM ROCK inhibitor (Y-27632). Add 500 μL medium per well.
39. Let the organoids recover for 3–4 days. Refresh the medium every 2 days.

**Figure 3:**
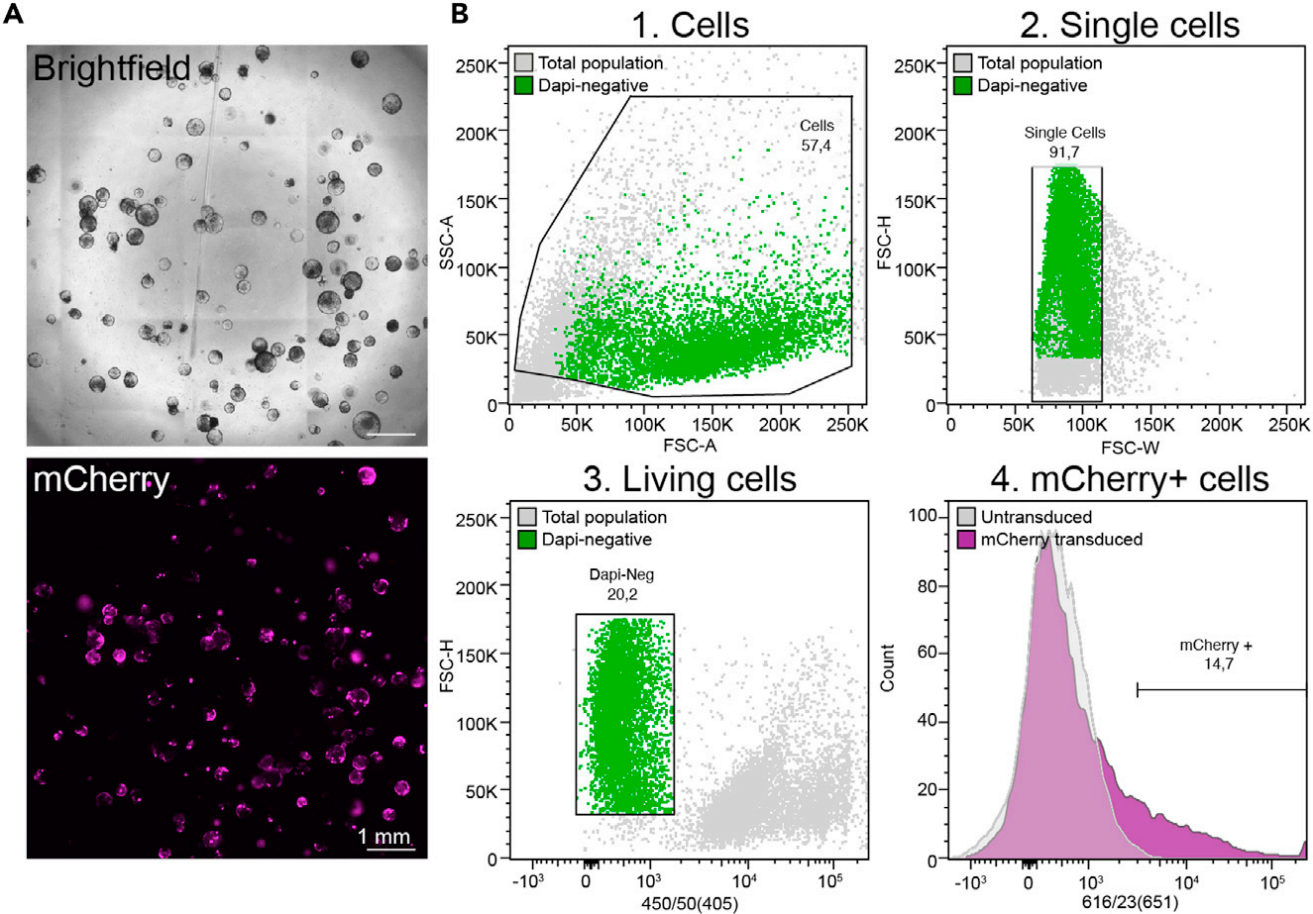
Transducing and sorting fluorescently labeled organoids (A) Full well microscopy images of unsorted mCherry transduced organoids on brightfield and mCherry channels, scalebar 1 mm. (B) Gating strategy for sorting mCherry-positive cells. From the FSC;SSC plot, the living (DAPI-negative) cells are visualized as a readily distinguishable population (depicted in green).

***Note:*** After a few days you can check for fluorescent signals under a fluorescence microscope. An example of a sample efficiently transduced with mCherry virus can be found in Figure 3A. If you estimate the percentage of positive cells to be >10%, you can expand your organoids and sort them using Fluorescent Activated Cell Sorting (FACS), 4–6 full wells of a 24-well plate should be sufficient for sorting. We aim to at least sort 1,000 positive cells, however, in our experience this experimental procedure will provide us between 10,000 and 100,000 positive cells.

***Alternatives:*** If your integrated fluorescent construct also contains an antibiotic resistance cassette, antibiotic selection with e.g. puromycin or blasticidin can also be used to enrich positive cells. However, we prefer to use a direct FACS-based approach as organoids can be particularly sensitive for antibiotic selection and thus careful titration of antibiotic agents should be performed before selection.

### Protocol C. Sorting the positive cells

To select for efficiently transduced organoids, organoids can be sorted based on their fluorescent label. This protocol describes the preparation of samples for FACS cell sorting and shows the sorting gating strategy.

**Timing: Steps 40–52: 1 h + 30 min. Steps 53–62 is dependent on the FACS sorting machine, flow rate and the percentage of positive cells.**

**CRITICAL:** All experiments involving lentiviral particles must be performed in a biosafety level 2 (BSL 2) lab. Please take care in following BSL 2 regulations on experimental handling and discarding of plastics, cells and media.

***Note:*** To increase the outgrowth potential of the organoids it is advised to culture the organoids in ENR medium containing 10 μM CHIR-99021 for at least 3 days prior to sorting to elevate their stem cell-like state. Please ensure you include at least 1 full well of a 24-well plate containing untransduced organoids to serve as a negative control to set your FACS gating.

40. Remove ENR medium from the wells containing organoids.
41. To disrupt the Matrigel containing the organoids, add 250 μL Cell Recovery Solution for every 50 μL Matrigel dome and mechanically disrupt the organoid structures by pipetting the suspension up and down using a P1000 filter tip.

***Note:*** Organoids from different wells in a 24-well plate can be pooled. Pipet approximately 10 times up and down for a proper breakdown into small organoid fragments. Scraping the well using the P1000 tip may be necessary to detach leftover Matrigel.

42. Transfer the organoid suspension to a sterile 15 mL tube and incubate on ice for 30 min.
43. In order to remove cell debris, centrifuge the tube for 4 min at 480×*g*, discard the supernatant.
44. To the cell pellet, add 250 μL TrypLE for dissociation to single cells.
45. For the optimal generation of a single cell suspension, incubate the solution at 37°C for 4 min.

**CRITICAL:** Generating a single cell suspension is critical to prevent sorting doublets, as some cells may not express the fluorophore. Furthermore, a single cell suspension is required to prevent the cell analyzer from clogging.

46. In the meantime, prepare the FACS buffer solution consisting of ice-cold 1% FCS/PBS.
47. After incubation at 37°C, add 1 mL of FACS buffer solution directly to the 15 mL tube containing the cell suspension to neutralize the TrypLE. Pipet up and down thoroughly.
48. Centrifuge for 4 min at 480×*g.*

***Note:*** Due to the dissociation of the cells, the cell pellet will appear smaller than in previous steps.

49. Remove supernatant and wash the cell pellet once more with 1 mL of the FACS buffer solution. Pipet up and down for approximately 5 times and centrifuge for 4 min at 480×*g.*

**CRITICAL:** After TrypLE treatment the cell pellet tends to be sticky and difficult to dissociate. If the pellet does not dissociate completely, add 1 mL fresh ice-cold FACS buffer, pipette thoroughly, and centrifuge again. Repeat this step until the pellet is completely dissociated.

50. Remove supernatant and add 250 μL FACS buffer to the cell pellet.
51. Transfer the cell suspension into FACS tubes.

***Note:*** Depending on density and the number of organoid wells used for cell sorting, the volume of FACS buffer to be added may differ. For 4–6 wells of organoids from a 24-well plate, 250 μL FACS buffer is sufficient.

52. Prepare collecting tubes for each sample containing 1 mL of pre-warmed ENR medium (37°C).
53. Directly before measuring your sample, add DAPI to a final concentration of 10 μM to distinguish living/dead cells. Samples are now ready for flow cytometry.

**CRITICAL:** This protocol describes sorting cells on the BD FACSAria III Cell Sorter, using a 85 μm nozzle and a flowrate between 200–3000 events/second. Nozzle size, flow rates, lasers and laser settings may differ on distinct cell sorters.

***Note:*** To set optimal laser voltages and to correctly gate your positive population, it is suggested to take an untransduced (negative control) sample along.

54. Determine the gating of your sample. An example of a gating strategy to sort for mCherry transduced organoids is presented in Figure 3B.
55. First, gate the cell population using the forward scatter (FSC)-A and side scatter (SSC)-A.
56. Next, the single cell population can be determined using the FSC-H and FSC-W plot.
57. To select for a living cell population, gate the DAPI-negative cell population using FSC-Height, a 405 nanometer (nm) laser and a 450/50 nm bandpass filter.
58. mCherry+ cells are selected using the 616/23(561) nm laser.
59. Once all settings have been established, sort as much positive cells as the sample contains.
60. After sorting, transfer the samples from the FACS collection tubes to a sterile 15 mL tube and centrifuge at 480×*g* for 4 min.
61. Add a sufficient amount of Matrigel (volume is dependent on the number of sorted cells and the number of wells to be plated). Generally, we plate 10.000 cells per 50 μL Matrigel per well in a 24-well plate.
62. Culture sorted organoids in ENR medium containing 10 μM CHIR-99021 and 10 μM ROCK inhibitor (Y-27632) until passaging to increase outgrowth potential.

***Note:*** Throughout this protocol, mCherry fluorescence (red) will be depicted in magenta to enhance readability and interpretation for the color-blind.

## Key resources table

**Table:** 

REAGENT or RESOURCE	SOURCE	IDENTIFIER
**Chemicals, peptides, and recombinant proteins**		

DMEM	Thermo Fisher Scientific	Cat#10566-016
OptiMEM	Thermo Fisher Scientific	Cat#31985-070
Advanced DMEM/F-12	Thermo Fisher Scientific	Cat#12634-028
Antibiotic-Antimycotic (100×)	Thermo Fisher Scientific	Cat#15240-062
N-2 Supplement (100×)	Thermo Fisher Scientific	Cat#17502-048
B-27 Supplement (50×), serum free	Thermo Fisher Scientific	Cat#17504-044
GlutaMAX Supplement	Thermo Fisher Scientific	Cat#35050-038
HEPES buffer (1M)	Thermo Fisher Scientific	Cat#15630-056
N-Acetyl-L-cysteine	Sigma-Aldrich	Cat#A9165
EGF Mouse Recombinant	tebu-bio	Cat#315-09
CHIR-99021	tebu-bio	Cat#10-1279-5mg
Y-27632 dihydrochloride (ROCK Inhibitor)	Sigma-Aldrich	Cat#Y0503
Corning Matrigel	Corning	Cat#356231
Polybrene	Sigma-Aldrich	Cat#107689
Cell Recovery Solution	Corning	Cat#354253
TrypLE Express Enzyme	Thermo Fisher Scientific	Cat#12604-013
Fetal Calf Serum (FCS)	Sigma-Aldrich	Cat#F7524
Phosphate Buffered Saline (PBS)	Fresenius Kabi	n/a
DAPI fluoro grade	Invitrogen	Cat#D21490
Collagen Rat Tail Type I	Corning	Cat#354236
Sodium Bicarbonate	Sigma-Aldrich	Cat#S8761
Sodium Hydroxide	Sigma-Aldrich	Cat#1310-73-2
Phosphate Buffered Saline (PBS) Tablets	Gibco	Cat#18912-014
Collagenase Type IV	Sigma-Aldrich	Cat#C5138-1G
Lipofectamine 2000	Thermo Fisher Scientific	Cat#11668-019
Cryogenic vial	Corning	Cat#431386

**Recombinant DNA**		

pMD2.G (envelope)	Addgene	Cat#12259
pMDLg/pRRE (packaging)	Addgene	Cat#12251
pRSV-Rev (packaging)	Addgene	Cat#12253
LeGO-C2	Addgene	Cat#27339
LeGO-V2	Addgene	Cat#27340

**Experimental models: Cell lines**		

HEK293T cells for production of viral particles (Protocol A)	n/a	n/a
HEK293T Noggin producing line	Laboratory of Hans Clevers, Hubrecht Institute	HEK293-mNoggin-Fc
HEK293T R-Spondin 1 producing line	Laboratory of Calvin Kuo, Stanford	293T-HA-RspoI-Fc
Murine small intestinal organoids	n/a	n/a

**Other**		

Greiner CELLSTAR 48 well culture plates	Greiner Bio-One	Cat#677180
Greiner CELLSTAR 24 well culture plates	Greiner Bio-One	Cat#662160
Greiner CELLSTAR 6 well culture plates	Greiner Bio-One	Cat#657160
5 mL FACS tubes	Corning	Cat#352008
BD Trucount Tubes	BD Biosciences	Cat#340334
Amicon Purification System with 100kDa Amicon Ultra-0.5 Device	Merck Millipore	Cat#ACS510024
0.2 μm filters	VWR	Cat#514-0073
0.45 μm filters	VWR	Cat#514-0075
50 mL syringes	BD Plastipak	Cat#10636531
15 mL tubes	Greiner Bio-One	Cat#188271
50 mL tubes	Greiner Bio-One	Cat#227261
10 cm plates	Greiner Bio-One	Cat#664960
P1250 Long Reach Filter Tips	Westburg	Cat#WS5074
P200 Long Reach Filter Tips	Westburg	Cat#WS5040
P20 Long Reach Filter Tips	Westburg	Cat#WS5020
P10 Long Reach Filter Tips	Westburg	Cat#WS5010
Panasonic CO2 incubator	Panasonic Healthcare corporation	MCO-170AICUVH-PE
Hettich ROTINA 420R swinging bucket centrifuge	Hettich	Z723754
BD LSRFortessa Cell Analyzer	BD Biosciences	n/a
BD FACSAria III Cell Sorter	BD Biosciences	n/a
EVOS Cell Imaging System	Thermo Fisher Scientific	Cat#AMAFD1000

**Software and algorithms**		

BD FACSDiva Software V8	BD Biosciences	n/a
FlowJo Software	BD Biosciences	n/a
ImageJ Software	https://imagej.nih.gov/ij/	n/a

## Materials and equipment

### Medium for organoid cultures (ENR)

To prepare the standard organoid medium see the table below. Standard organoid medium can be kept at 4°C for up to 2 months or alternatively can be aliquoted in 50 mL tubes and stored at −20°C for up to 6 months.

Organoids are cultured in standard organoid medium freshly supplemented with growth factors mEGF (E), Noggin (N), and R-spondin1 (R), which we will refer to as ‘ENR’ medium. A detailed protocol on the production of Noggin and R-spondin1 conditioned medium from producer lines can be found elsewhere (Fujii et al., 2015; Vonk et al., 2020). ENR medium is prepared freshly before adding it to the organoid cultures in the following concentration (Store for maximum of 7 days at 4°C):

• 70% organoid medium (Table 2)
• 20% R-spondin1 medium
• 10% Noggin medium
• 50 ng/mL mEGF

***Alternatives:*** Instead of in-house production of R-spondin1 and Noggin conditioned medium using a producer line, also recombinant R-spondin1 (R&D Systems, #7150-RS) and Noggin proteins (R&D Systems, #6997-NG) can be used.

### Additional medium components for Collagen cultures (steps 22, 34)

Add 10 μM Y-27632 (ROCK inhibitor) to the ENR medium during the first 3 days of plating organoids on the collagen matrix.

### Preparation of Collagen neutralization buffer (1 mg/mL)

Example: prepare a Collagen I solution from a stock concentration of 3.5 mg/mL to a final concentration of 1 mg/mL in 1 mL (Table 3).

**Table 3:** Components of collagen neutralization buffer

Reagents	Storage	Final concentration	Volume used
Sodium bicarbonate	RT	53 mmol/L	6 mL
HEPES	4°C	20 mmol/L	2 mL
NaOH (1N)	RT		575 μL
PBS (10×)	RT	1×	10 mL
H_2_O (DEMI- sterile)	RT		52.925^a^ mL
Total	4°C		71.5 mL

Protocol derived from Wang et al. (Wang et al., 2017).

a H_2_O = 71.5 mL–18.575 mL = 52.925 mL.

Collagen volume required for one reaction (1 mg) = 0.285 mL of a 3.5 mg/mL stock

Volume of Buffer required for neutralization for one reaction = 1 mL–0.285 mL = 0.715 mL

To prepare neutralization buffer for 100 reactions = 0.715 mL ∗ 100 = 71.5 mL

**CRITICAL:** Filter the neutralization buffer solution using 0.2 μm filters and store at 4°C for up to six months.

***Note:*** The volume of H_2_O and the volume of Neutralization buffer added to Collagen I depends on the stock concentration of Collagen I. This will vary from batch to batch.

### FACS buffer

Dilute 500 μL FCS in 49.5 mL PBS to a final concentration of 1% FCS in PBS. Store at 4°C for up to one week.

### DAPI

Stock solution (20 mM): add 1.4 mL ddH_2_O to 10 mg DAPI, aliquot and store at −20°C. Stock solution is stable for 1 year after preparation.

Working solution (200 μM): dilute stock solution 1:100 in ddH_2_O, store at 4°C. Working solution is stable for 2 months after preparation.

### PBS (10×)

Dissolve 1 tablet in 1 L of Distilled water. Sterilize by autoclaving for 20 min at 15 psi.

### Collagenase type IV

Dissolve 10 mg of Collagenase Type IV powder in 1 mL of sterile PBS (1×). Filter using 0.2 μm filters and place at 4°C for direct use or at −20°C to store for up to 1 year. Avoid multiple freeze-thawing events.

## Step-by-step method details

### Protocol 1: preparation of 3D organoid co-cultures

**Timing: 1 h**

This protocol describes the generation of 3D organoid co-cultures in Matrigel domes. To study cell competition *in vitro*, fluorescently labeled organoid populations can be cultured simultaneously in one well. This protocol provides detailed steps on how to initiate organoid co-cultures.

**CRITICAL:** For the interpretation of the co-culture results, it is critical to always take along a ‘control’ co-culture consisting of normal (wild type; WT) organoids in 2 distinct fluorescent colors (WT/WT) to compare with your experimental co-culture (WT/Mutant).

Before you start: Pre-warm 24-well culture plates at least 1 day prior to co-culture initiation.

**Figure 4:**
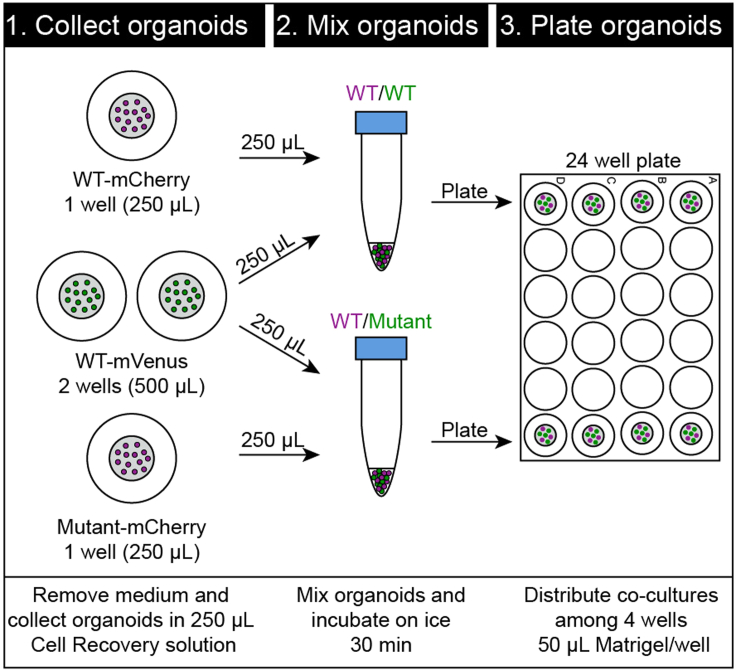
Example of 3D co-culture workflow in a 24-well plate generating a control WT/WT and experimental WT/Mutant co-culture with 4 technical replicates
3. Remove ENR medium from the wells containing organoids.
4. To take organoids out of the Matrigel, add 250 μL Cell Recovery Solution to 50 μL Matrigel domes (volume can be adapted according to the amount of domes) and forcefully disrupt the Matrigel by pipetting up and down using a P1000 filter tip.
5. Transfer the organoid solution of both populations in the same sterile 15 mL tube and pipet thoroughly up and down to mix and break down organoid fragments.
6. Incubate on ice for 30 min.

***Note:*** Depending on your research question and the clonogenicity, expansion rates, and expected competitive effects (advantage/disadvantage) of your organoids, different plating ratios (Figure 5) can be used.

1. Check well confluency and organoid condition under the microscope to assess whether they are suited for co-culture experiments. An example of optimal organoid density within a 24-well plate is presented in Figure 2A.
2. To generate co-cultures in a 1:1 ratio, select wells with similar organoid density as estimated by eye. Alternatively, the amount of organoids per well of a 24-well plate can be counted by manual counting or by scanning the well in its fluorescent channel and quantifying using ImageJ. An example of a plating strategy in a 24-well plate with 4 technical replicates is given below (Figure 4)

7. After incubation on ice, centrifuge the tube at 480×*g* for 4 min in order to remove cell debris. Discard the supernatant.

**Figure 5:**
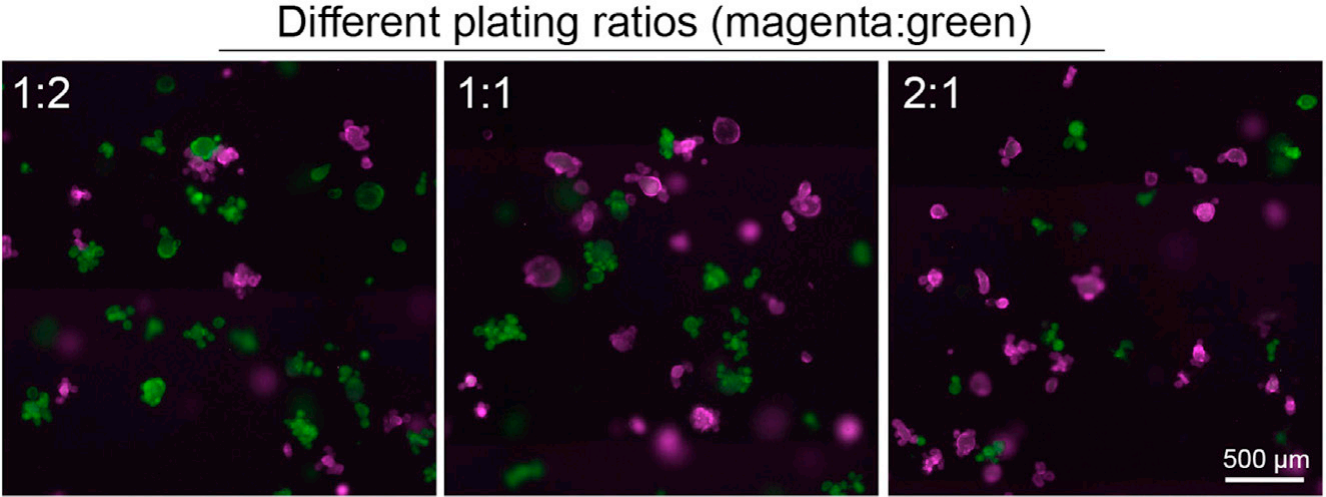
Co-cultures of organoids using different (magenta:green) plating ratios, scalebar 500 μm
8. Resuspend the organoid pellet in an appropriate volume of Matrigel (volume dependent on the amount of co-culture wells to be generated).

***Note:*** 50 μL of Matrigel per seeded well of a 24-well plate is required for an optimal seeding density. It is recommended to add approximately 30 μL extra Matrigel to the total volume to ensure a sufficient amount of material available and to prevent the formation of bubbles. Essential in seeding co-cultures is to keep the Matrigel ice-cold for the duration of the handling.

9. Plate exactly 50 μL of Matrigel containing co-culture suspension per well of a pre-warmed 24-well plate. In between plating the wells, pipet the solution up and down to mix organoid populations.
10. Carefully transfer the culture plate to an incubator (37°C, 5% CO_2_) and leave the plate for approximately 15 min to solidify the Matrigel domes.

***Note:*** After approximately 5 min, turn the plate upside down for optimal distribution of organoid within the Matrigel dome and to prevent attaching to the plate’s surface.

11. Provide each well containing the organoid co-cultures with 500 μL of pre-warmed ENR culture medium.
12. Place organoids in a humidified incubator (37°C, 5% CO_2_).

***Optional:*** 3D co-cultures can also be expanded as regular cultures to enable analysis over several passages.

**Figure 6:**
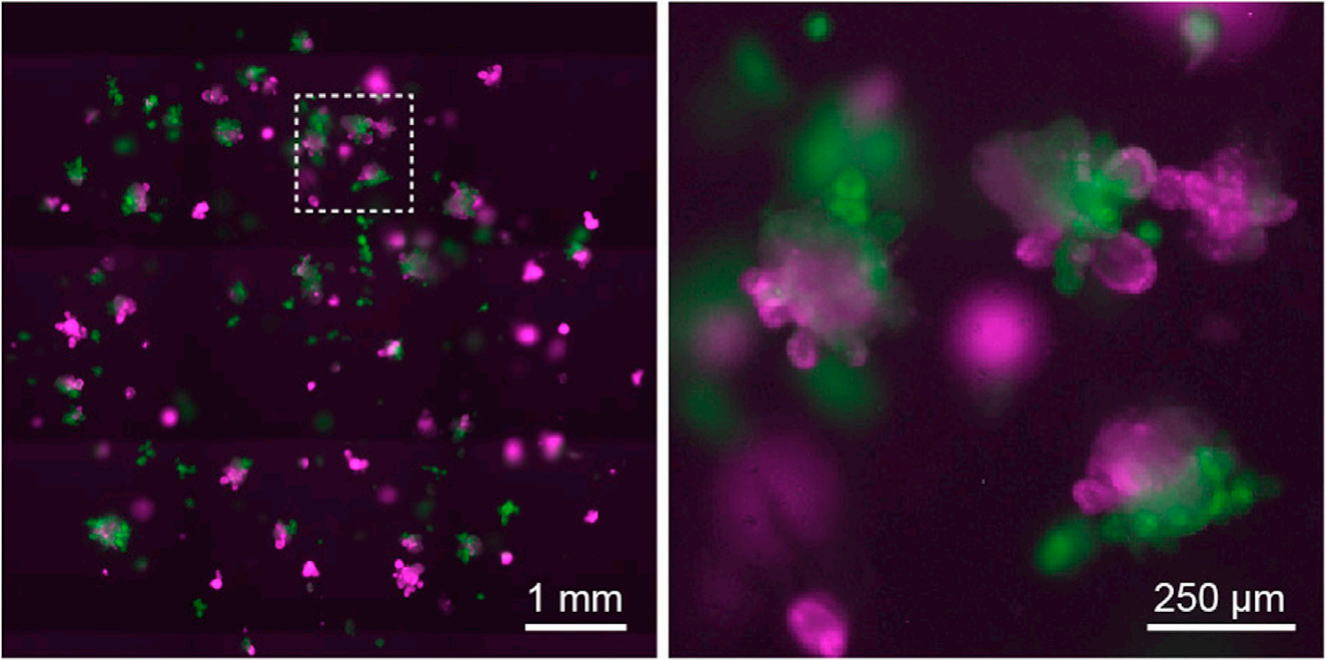
Example of co-culture with fused magenta:green organoids, scalebar 1 mm (left panel), 250 μm (zoom panel)

***Alternatives:*** To study contact dependent interactions in 3D, fused organoid co-cultures can be established. To generate fused magenta:green organoid co-cultures, mechanically disrupt organoids and mix suspensions in Eppendorf vials. Mildly centrifuge and resuspend the cell pellet in a small volume of ENR medium. Incubate the organoid mixtures at 37°C for 30 min to ensure cell fragments to stick together before centrifuging the organoid mixture at 480×*g* for 4 min, discarding the supernatant and plating the organoids in Matrigel domes (Figure 6). An excellent manuscript demonstrating complete details on the use and execution of this co-culture method, please refer to (Krotenberg Garcia et al., 2021).

### Protocol 2: preparation of 2D organoid co-cultures

This protocol describes the generation of 2D co-cultures of mouse intestinal organoids on collagen hydrogels. This protocol has three distinct steps: the preparation of the hydrogels, the establishment of 2D monocultures, and the generation of 2D co-cultures.

### Preparation of collagen hydrogels

**Timing: 1 h + 30 min**

For complete details on the use and execution of this part of the protocol, please refer to the step-by-step protocol from Wang et al. (Wang et al., 2017).

***Note:*** Prepare neutralization buffer for Collagen Rat Tail Type I (Table 3). Collagen hydrogel formation is dependent on pH and temperature: 1 mL of properly neutralized Collagen at 1 mg/mL will form a gel within 30–60 min at 37°C. Calculate the volume of Collagen I and neutralization buffer needed for a final concentration of 1mg/mL in 1 mL. For an example see Table 3.

13. Gently mix the calculated volume of Collagen I and the Neutralisation buffer.
14. Pipet the 1 mL on top of one well of a 6 well plate.
15. Incubate for 1 h at 37°C.

***Note:*** Plates can be prepared and stored up to one month at 37°C with 1 mL of PBS. Make sure to either refresh the PBS or seal the plates with parafilm to avoid hydrogels from drying out.

**Figure 7:**
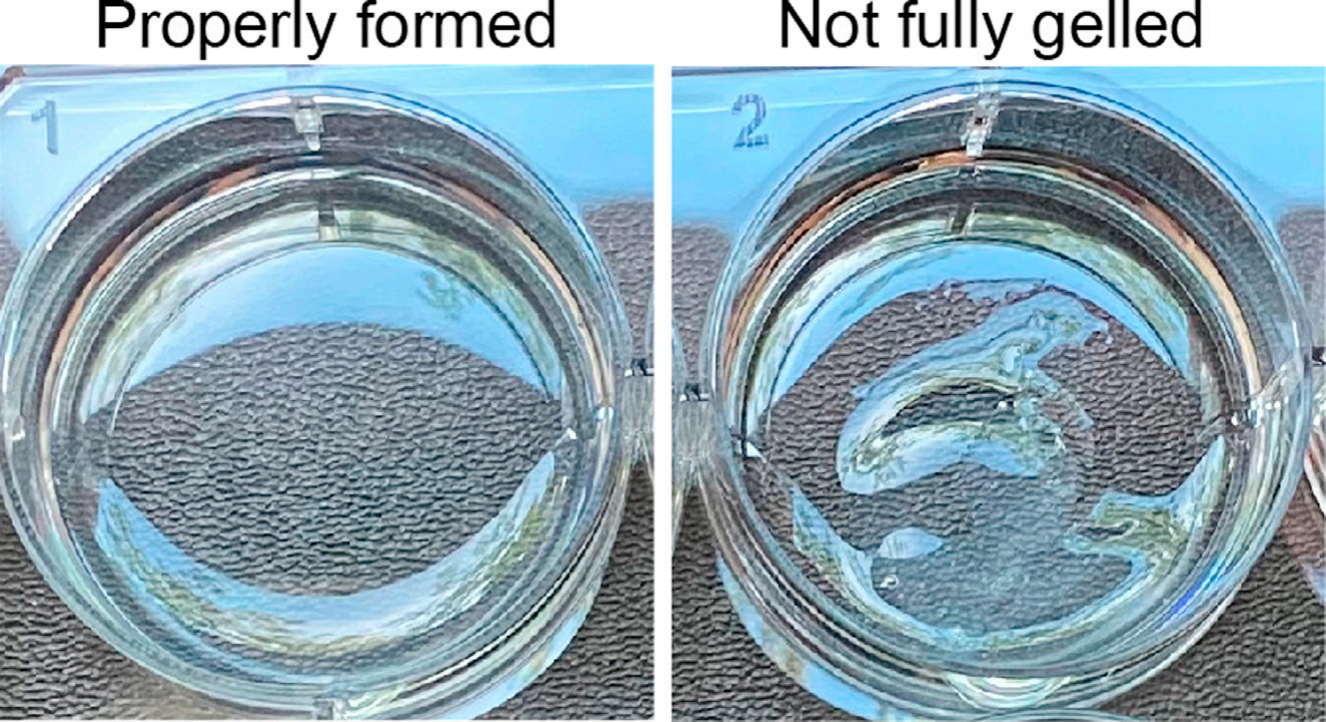
Examples of properly (1) and improperly (2) formed Collagen Hydrogels in a 6-well plate

**CRITICAL:** The Collagen hydrogel formation is critical for the cells’ growth. Examples of properly and improperly formed hydrogels can be found in Figure 7.

***Alternatives:*** 12 well plates can also be used for 2D Collagen cultures. Use 500 μL of neutralized Collagen solution for each well.

### Establishing collagen monocultures

For complete details on the use and execution of this part of the protocol, please refer to the step-by-step protocol from Wang et al. (Wang et al., 2017).

**Timing: 6 days**

***Note:*** Prepare Collagen hydrogels, and pre-warm ENR culture medium and PBS.

#### Day 1

**Timing: 1 h**

**CRITICAL:** Organoids are previously labeled with distinct fluorescent proteins.

16. Check well confluency and organoid condition under the microscope. For an example of optimal organoid density see Figure 2A.
17. Remove the ENR medium from the wells containing organoids.
18. Collect Matrigel containing the organoids in 250 μL of Cell Recovery Solution. Pipet the mixture vigorously up and down to break organoids apart.

***Alternatives:*** If pipetting is not sufficient in this step, organoids can also be broken apart by incubating with TrypLE for 5 min at 37°C.

19. Transfer the solution to a sterile 15 mL tube and incubate on ice for 30 min to dissolve Matrigel.

***Note:*** 2 full wells of organoids grown in 50 μL of Matrigel in a 24-well plate can be used for 1 well of a 6-well plate for collagen cultures.

20. After incubation, centrifuge at 480×*g* for 4 min.
21. Remove supernatant and continue with the cell pellet.

***Optional:*** If residual Matrigel is visible, add 1 mL of ice-cold PBS and repeat steps 20 and 21.

22. Resuspend pellet in 2 mL organoid medium supplemented with ENR and ROCK inhibitor.
23. Gently pipet the cell solution on top of the Collagen hydrogel.

**CRITICAL:** Pipet the mixture drop by drop to avoid breaking the Collagen hydrogel.

#### Day 3 & 5

**Timing: 10 min**

***Note:*** Pre-warm ENR culture medium.

24. Check the cells under the microscope to make sure they are attached and growing on the hydrogels.
25. Gently aspirate the old medium and add 2 mL of new ENR culture medium.

***Note:*** Cells grown on Collagen hydrogels can be split every 5–7 days. Check the confluency well under the microscope. Once the plate is at 80% proceed to passaging (steps 26–33). 1 well of a 6-well plate can be used for 2/3 new wells of a 6-well plate depending on confluency.

### 2D co-culture assay

**Timing: 6 days**

***Note:*** Prepare collagen hydrogels, and pre-warm ENR culture medium and PBS.

***Note:*** 3D cultures take time to adapt to the new 2D environment, for this reason it is advised to grow the organoids first as monocultures on Collagen I hydrogels for at least 1 passage before proceeding to co-cultures.

#### Day 1

26. Collect the Collagen hydrogels and the 2D grown cells using a 5 mL stripette with 1 mL PBS and transfer the mixture in a 15 mL tube.

***Note:*** For co-culture experiments, choose two lines with different fluorescent labels.

27. Add 50 μL of Collagenase Type IV and place in the incubator at 37°C for 10 min.
28. After incubation, add 10 mL of PBS at RT and pipet vigorously. The large volume of PBS will help to dilute out any remaining collagenase.
29. Centrifuge at 480×*g* for 4 min.
30. Remove supernatant.
31. Repeat steps 28–30 two times.
32. Resuspend the pellet in 1 mL ADF medium supplemented with ENR.
33. Count cells using a cell counting chamber.
34. Mix equal amounts of the two lines (50:50) in an organoid medium supplemented with ENR and ROCK inhibitors to a final volume of 2 mL. The cell seeding density will depend on the plate used (Table 4).

**Table 4:** Cell seeding density

Plate used	Total cell seeding number
6 well plate	0.3 × 10^ˆ6^ cells
12 well plate	0.15 × 10^ˆ6^ cells

***Note:*** The table provided gives an indication for the use of wild type organoids. If using organoids other than wild type, in which proliferation rate might differ, adjust accordingly.

***Alternatives:*** Other ratios of each line can be used depending on the experimental aim.

35. Pipette vigorously to homogenize solution
36. Gently pipette mixture on top of a Collagen well.

***Optional:*** If performing a co-culture assay with drug treatment, add your drug at the desired concentration to the culture medium and refresh according to the properties of the drug.

At predetermined timepoints:

37. Fluorescent microscopy imaging is used to analyze the size and growth of cells on the 2D co-culture.

***Optional:*** Flow cytometry analysis can also be performed for 2D cocultures. In this case, prepare separate wells for different timepoints.

**CRITICAL:** Washing steps are very important to remove residual Collagenase. Remnants of Collagenase can deteriorate the Collagen hydrogels.

***Note:*** Medium is changed every other day with a new ENR medium.

### Protocol 3. Co-culture imaging and analysis

#### Microscopy imaging

39. Place your organoid co-culture plate in the imaging system.
40. Bring your cells into focus.

***Note:*** To choose a focus point for 3D organoid co-cultures, go through the z planes of one well and select the plane containing most organoids in focus. Keep the same focus for all wells.

41. Optimize fluorescent intensity of all required channels separately.
42. Create your own scan routine, depending on the microscope used.
43. Scan and save your files.

**CRITICAL:** Keep laser intensity settings equal between all conditions and for different timepoints. Focus on your co-culture using only one and the same color at every timepoint.

***Note:*** The imaging for this protocol was performed using an EVOS FL Auto Imaging System using GFP (excitation 470/22; emission 525/50) and Texas Red (excitation 585/29; emission 628/49) LED light cubes. Using the EVOS system, overexposed areas will turn red on the screen. For optimal fluorophore detection and analysis, set your laser intensity just below this overexposure detection. The laser intensity and detection of overexposed areas can differ depending on the microscope used.

#### Image analysis

**Figure 8:**
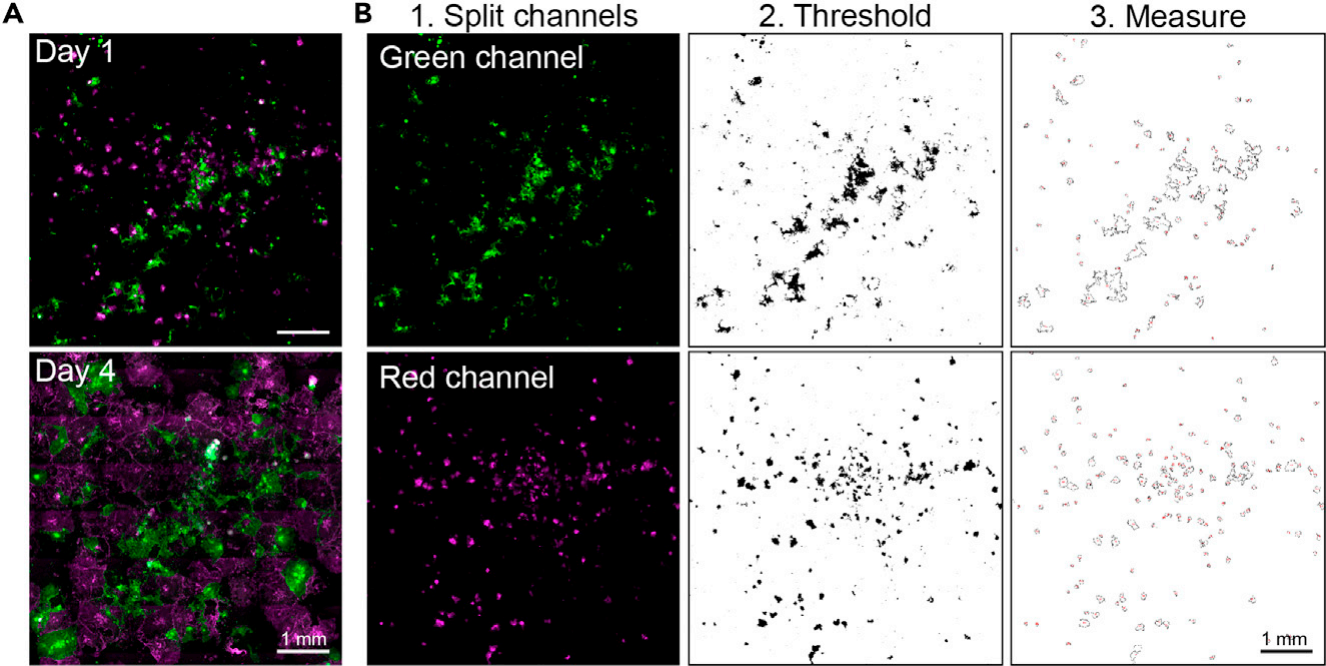
Imaging and analysis of 2D co-cultures (A) Example of expansion of 2D co-culture on collagen over time, scalebar 1 mm. (B) Image analysis pipeline of Day 1 co-culture consisting of (1) splitting fluorescence channels, (2) setting a thresholding cutoff, and (3) measuring individual particles, scalebar 1 mm.

**Figure 9:**
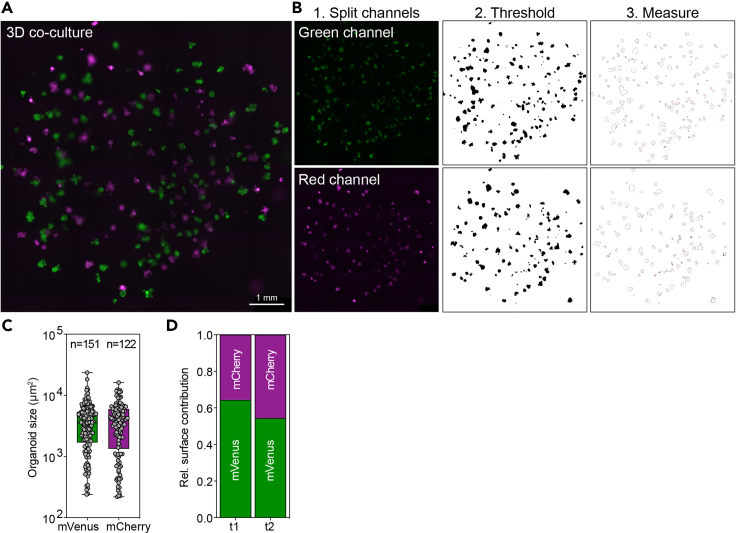
Imaging and analysis of 3D co-cultures (A) full well image of 3D organoid co-culture, scalebar 1 mm. (B) Image analysis pipeline consisting of (1) splitting fluorescence channels, (2) setting a thresholding cutoff, and (3) measuring individual particles, scalebar 1 mm. (C) Average organoid number and size can be quantified per timepoint. Boxplot visualizes minimum to maximum value, each dot represents an organoid. (D) Relative surface contribution can be measured over several timepoints (example).

The image analysis for the 2D and 3D co-cultures is performed using ImageJ software. Using ImageJ, the number and size of the organoids (for 3D co-cultures) and the surface occupation (for 2D and 3D co-cultures) can be determined. Instructions and examples of image processing and analysis for 2D (Figure 8) and 3D (Figure 9) co-cultures are discussed below. For extensive user and troubleshooting guides, please refer to the ImageJ website (www.imagej.nih.gov/ij/docs/guide/146.html).

44. Open ImageJ software
45. Import the scanned images
46. In the top browser open Image → Color → Split Channels

***Note:*** Keep the channels based on your fluorophore open and close the channels you won’t use for the analysis. Each channel is now a single image with pixel values between 0 and 255 (for 8-bit images) which can be used for thresholding.

47. Open Image → Adjust → Color Balance
48. Set display range for each individual channel

***Note:*** Display range will depend on laser settings. If settings were kept equal for both lasers while scanning then display range settings should be similar.

49. Open Image → Adjust → Brightness and contrast
50. Set display range for individual channel

***Note:*** Adjusting the lower and upper limits of the display range helps to convert the informative pixels within the optimal range of 0–255. For example, adjusting the lower limit can exclude autofluorescent background signals. In addition, some fluorescent artefacts sometimes define the outer limit, which can be adjusted to the brightest true fluorescent signal. After setting the lower and upper limit, the pixel values of 0–255 will be mapped to the new display range.

51. Open Image → Adjust → Threshold and set the threshold range

***Note:*** Defining a threshold converts the pixels into binary classes, e.g. positive/negative or background/foreground. In our experiments, a threshold was used to separate the fluorescent areas from the background. Several images need to be checked so that the threshold only covers the fluorescent areas. There are various threshold algorithms to choose from, and the one that fits best to detect the fluorescent signal should be used.

**CRITICAL:** Keep the threshold algorithm and threshold values equal between all experimental conditions. For the analysis below, the ‘Default’ threshold has been used (Table 5).

52. Open Analyze → Analyze Particles:

**Table 5:** Particle analysis settings

Size	100-Infinity
Circularity	0.00–1.00
Show	Outlines

***Note:*** ‘Size’ is the minimum number of pixels an object should have to be measured as an object. For our experimental 2D and 3D approaches, exclusion of measurements with <100 pixels results in significant noise reduction without loss of experimental data. The function ‘circularity’ includes or excludes objects depending on their shape, with 0.00 being the least circular and 1.00 being a perfect circle. As in our co-cultures experiments 3D organoids and 2D layers can have any possible shape, circularity function will not be used. Depending on your research question these parameters can be adapted accordingly. The ‘show’ function can be utilized to visualize the measurements being made and to validate whether the determined parameters do not lead to data loss.

53. Tick the box “display results” for a table containing area measurements.
54. Copy/store the Area measurements and paste them in an excel sheet for analysis.

**CRITICAL:** Using the abovementioned protocol, area measurements will be given in pixels, which is sufficient when determining the relative surface contribution. However, to translate this data to the absolute size and surface expansion (in e.g. μm^2^) convert the pixel area to μm^2^. Depending on the imaging system used, the pixel-to- μm^2^ ratio is already included or can be manually entered via: analyze → set scale.

### Protocol 4. Co-culture flow cytometry and analysis

**Timing: 1 h + 30 min**

To analyze the relative abundance of the organoids in the co-culture system, flow cytometry analysis can be performed. Organoid cultures are fluorescently labeled which enables tracing of co-culture contributions.

***Optional:*** When essential to determine the absolute cell numbers within a well, BD Trucount FACS tubes (BD Biosciences) can be used. These tubes contain a fixed number of beads that can be easily distinguished from your cell population in the FSC-A/SCC-A plot.

**Figure 10:**
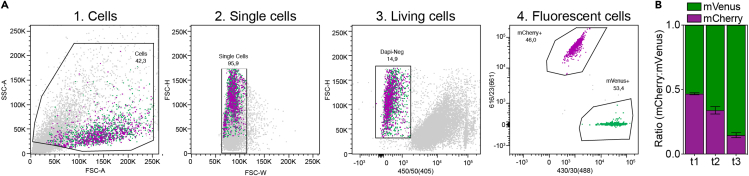
FACS analysis of 3D co-culture (A) Gating strategy to determine the ratio of mCherry:mVenus cells. (B) Example of co-culture analysis measured over several timepoints (*t*). Data are mean ± SD.

55. To prepare samples for flow cytometry analysis, refer to steps 40–51 of the ‘Sorting the organoids’ protocol described in Protocol C.
56. Set up your gating strategy using samples that contain a single fluorophore. An example of a co-culture gating strategy to analyze co-cultures of mCherry and mVenus transduced organoids is presented in Figure 10.

**CRITICAL:** This protocol describes flow cytometry analysis on the BD LSRFortessa Cell Analyzer, using a flow rate between 100–1000 events/second. Flow rates, lasers and laser settings may differ on distinct cell analyzers.

57. Directly before measuring your sample, add DAPI with a final concentration of 10 μM to distinguish living/dead cells.
58. First, gate the cell population using the forward scatter (FSC)-A and side scatter (SSC)-A.
59. To exclude doublets, use FSC-H and FSC-W density plots.
60. To select for a living cell population, gate the DAPI-negative cell population using FSC-H and 405 nanometer (nm) laser and a 450/50 nm bandpass filter.
61. To calculate the ratio of mCherry+ to mVenus+ cells, mCherry+ cells are detected using the 612/23(561) nm laser and mVenus cells are measured using a 530/30(488) nm laser.

## Expected outcomes

The 2D and 3D co-culture approaches proposed in this protocol are ultimately developed to study interactions between normal (wild type; WT) and mutant organoids. Based on the experimental readout, either by flow cytometry or microscopy, information can be acquired regarding cell numbers, organoid size and shape. In addition, relative expansion rates or surface contribution can be measured over time. In order to correctly interpret the results of the proposed co-cultures it is essential to always include a bi-colored WT/WT co-culture as a control to the WT/Mutant co-culture. It is expected that the WT organoids in the control co-culture have equal expansion rates, and therefore the relative ratios of WT/WT co-cultures should remain equal as well. For the experimental co-culture, the relative ratio can be anywhere between 0–1, depending on individual expansion rates and/or the interaction between WT/Mutant organoids.

### Expected outcomes: flow cytometry

The example illustrated in Figure 10 shows a change in the ratio of mCherry:mVenus cells in favor of the mVenus population as measured by flow cytometry at three distinct timepoints. This result can be explained by (1) different growth rates between the two populations or (2) active competition between the two populations e.g., one population inhibits the expansion of the other.

One example to test these two options is by analyzing your samples in TruCount FACS tubes. Due to the fixed number of beads contained in the TruCount tubes, they give an estimate of the absolute number of fluorescently labeled cells over time and thus their expansion rates. Whenever the expansion rates of mCherry+ cells between the control and experimental co-culture stay equal over time, the difference in mCherry:mVenus ratio is highly likely the result of different growth rates. This can be validated using e.g., nucleotide incorporation assays. However, when the expansion rates of mCherry+ cells decrease in the experimental co-culture compared to the control culture, this could imply an active inhibitory effect on the mCherry+ population by the mVenus+ population. Analyzing your co-cultures using both flow cytometry and microscopy can also give you visual indications and measurements of expansion rates that can already point towards either one of the explanations.

## Limitations

This protocol describes the successful establishment of 3D and 2D coculture assays of intestinal organoids. However, several limitations are implied; imaging 3D cultures can be challenging due to the presence of several superimposed planes, causing a focusing problem. The use of 2D cultures provides an alternative to overcome this problem.

Another imaging limitation is caused by the accumulation of dead cells, either on the luminal side of 3D organoid cultures or on the top surface of 2D cultures. Dead cells can contribute to autofluorescence due to the presence of extracellular matrix debris and other excreted proteins. Autofluorescence can be reduced by adjusting the laser intensity before performing a scan. On another hand, flow cytometry quantification, using DAPI stain to separate live from dead populations, can help overcome this imaging limitation.

Furthermore, the manual analysis of the microscopy images using ImageJ can be tedious due to the time it takes to find the threshold that fits your experiment and analyze all images one by one. As determining the optimal threshold can also suffer from user bias, it is critical to maintain fixed settings for both microscopy lasers and image analysis to reduce variability between users and experiments.

The high volume of Matrigel and Collagen needed for technical and biological replicates can become costly; Matrigel can be diluted down to 7 mg/mL of the original concentration. In addition, you can scale down your experiment from a 24 to a 48-well plate (3D cultures), and from a 6 to a 12-well plate (2D cultures) to reduce costs.

Finally, the use of organoids to reconstruct *in vivo* tissue dynamics is limited due to the passaging requirements of organoid cultures. 2D and 3D organoid cultures need to be passaged approximately once a week (this time can be extended by passaging smaller organoid fractions). As *in vivo* tissue dynamics are an ongoing, uninterrupted entity, it is important to validate your *in vitro* findings in an *in vivo* model.

## Troubleshooting

### Problem 1

Low viral transduction efficiency (before you begin the section).

### Potential solution

If transduction efficiency is poor (< 10% positive cells), there are several optimization steps that can be applied during the generation of the viral particles, and the transduction of the organoids.

#### Generation of viral particles

First check whether your plasmid of choice should be packed using 2^nd^ or 3^rd^ generation viral packaging plasmids. The proposed protocol describes packaging of 3^rd^ generation plasmids, hence 2^nd^ generation plasmids cannot be packed using the described packaging vectors.

Check the confluency of your 293T cells, they should be ∼70% confluent at the start of transfection. Too sparse or too dense plated cells will result in suboptimal virus production.

Quality of the virus should also be maintained by proper storing conditions (−80°C) and avoiding freeze-thaw cycles by generating aliquots. Frequently thawing reduces viral quality and should therefore be avoided.

To test whether viral particles are produced, it is advised to test transduction of a cell line that is easy to transduce, such as 293T cells.

#### Transduction of the organoids

After you’ve confirmed efficient virus production, transduction of organoids can be further optimized.

If organoids are not properly cystic after 3 day incubation with CHIR-99021, increase the duration of the treatment. Organoids can even be passaged in the presence of CHIR-99021.

If organoids do not survive the spinfection followed by overnight incubation in the absence of Matrigel, try to plate organoids in Matrigel earlier, e.g., 4 h after spinfection.

It might be useful to titrate the amount of viral particles per well, it could simply be that you need more particles per reaction.

### Problem 2

Organoids are not growing in 3D in Matrigel (Protocol 1, step 9–10)

### Potential solution

To overcome the problem of organoids not growing in 3D using Matrigel, it is recommended to flip the plate upside down until the Matrigel solidifies as described in this protocol. If the plate is not flipped upside down, the Matrigel domes can collapse causing the organoids to stick to the plate bottom and grow in 2D. In addition, if organoids are plated too dense, they will not have enough physical space and nutrients to efficiently grow out.

### Problem 3

Difficulties maintaining consistent ratios of 3D co-cultures over multiple experiments. (Protocol 1, step 1–9)

### Potential solution

By passaging your organoids consistently over multiple passages, your cultures will contain approximately similar numbers of organoids per well per passage. If so, your 3D co-cultures tend to remain reproducible as well. If this is not the case, it is advised to count the number of organoids per well before starting your co-culture experiment and adapt the number of organoids to be included in your co-culture accordingly. Organoids can either be counted manually using a hand-held cell counter, or full wells can be scanned using a microscope and organoids can quickly be counted using ImageJ software.

### Problem 4

Collagen won’t form a gel after 1 h incubation at 37°C. (Protocol 2, step 15)

### Potential solution

Always store the neutralization buffer at 4°C. Make sure that the neutralization buffer is working properly by testing the pH (7–7.4) and that you are mixing the correct amount of Collagen to the buffer. When mixing, pipet gently up and down. Avoid shaking the plate in the first few minutes of gel formation. In our experience, preparing volumes up to 2 mL of neutralized collagen mixtures works best. Larger volumes can cause the collagen to precipitate during the neutralization step.

If collagen hydrogels are still not forming properly, check that all the concentrations are calculated correctly and prepare a new neutralization buffer if needed.

### Problem 5

Collagen hydrogel dissolves when medium and cells are added on top. (Protocol 2, step 23)

### Potential solution

Pre-warm the medium before use. Pipet the mixture gently, drop by drop, on top of the collagen plate. Make sure all washing steps are completed for the removal of collagenase IV, remnants can disrupt the formed hydrogel.

### Problem 6

Difficulties setting up the display range or threshold for image analysis on ImageJ. (Protocol 3, step 48–50)

### Potential solution

We recommend to resort to the ImageJ website for detailed user guides, tutorials and tips and tricks to set up the most accurate analysis (www.imagej.nih.gov/ij/docs/guide/146.html).

## Resource availability

### Lead contact

Further information and requests for resources and reagents should be directed to and will be fulfilled by the lead contact, Louis Vermeulen (l.vermeulen@amsterdamumc.nl).

### Materials availability

This study did not generate new unique reagents.

## Data Availability

This study did not generate data or code.

## References

[bib2] De Van Lidth Jeude J.F., Vermeulen J.L.M., Montenegro-Miranda P.S., Van Den Brink G.R., Heijmans J. (2015). A protocol for lentiviral transduction and downstream analysis of intestinal organoids. J. Vis. Exp. JoVE.

[bib1] Fujii M., Matano M., Nanki K., Sato T. (2015). Efficient genetic engineering of human intestinal organoids using electroporation. Nat. Protoc..

[bib3] Krotenberg Garcia A., Fumagalli A., Le H.Q., Jackstadt R., Lannagan T.R.M., Sansom O.J., van Rheenen J., Suijkerbuijk S.J.E. (2021). Active elimination of intestinal cells drives oncogenic growth in organoids. Cell Rep..

[bib4] Naujok O., Lentes J., Diekmann U., Davenport C., Lenzen S. (2014). Cytotoxicity and activation of the Wnt/beta-catenin pathway in mouse embryonic stem cells treated with four GSK3 inhibitors. BMC Res. Notes.

[bib6] Ramadan R., Van Neerven S.M., Wouters V., Garcia M., Muncan V., Franklin O., Battle M., Carlson K., Leach J., Sansom O. (2021). The extracellular matrix controls stem cell specification and tissue morphology in the developing and adult gut. bioRxiv.

[bib8] Sato T., Stange D.E., Ferrante M., Vries R.G.J., Van Es J.H., Van Den Brink S., Van Houdt W.J., Pronk A., Van Gorp J., Siersema P.D., Clevers H. (2011). Long-term expansion of epithelial organoids from human colon, adenoma, adenocarcinoma, and Barretts epithelium. Gastroenterology.

[bib7] Sato T., Vries R.G., Snippert H.J., van de Wetering M., Barker N., Stange D.E., van Es J.H., Abo A., Kujala P., Peters P.J. (2009). Single Lgr5 stem cells build crypt-villus structures in vitro without a mesenchymal niche. Nature.

[bib9] Schwank G., Clevers H. (2016). CRISPR/Cas9-Mediated genome editing of mouse small intestinal organoids. Methods Mol. Biol..

[bib5] van Neerven S.M., de Groot N.E., Nijman L.E., Scicluna B.P., van Driel M.S., Lecca M.C., Warmerdam D.O., Kakkar V., Moreno L.F., Vieira Braga F.A. (2021). Apc-mutant cells act as supercompetitors in intestinal tumour initiation. Nature.

[bib10] Vonk A.M., van Mourik P., Ramalho A.S., Silva I.A.L., Statia M., Kruisselbrink E., Suen S.W.F., Dekkers J.F., Vleggaar F.P., Houwen R.H.J. (2020). Protocol for application, standardization and validation of the forskolin-induced swelling assay in cystic fibrosis human colon organoids. STAR Protoc..

[bib11] Wang Y., DiSalvo M., Gunasekara D.B., Dutton J., Proctor A., Lebhar M.S., Williamson I.A., Speer J., Howard R.L., Smiddy (2017). Self-renewing monolayer of primary colonic or rectal epithelial cells. CMGH.

